# A Survival Case of High-Dose Amlodipine Intoxication With Unusual Manifestation of Type 2 Respiratory Failure

**DOI:** 10.7759/cureus.80768

**Published:** 2025-03-18

**Authors:** Nicholsan Jesiah, Yathukulan Siva, Pakkiyaretnam Mayurathan

**Affiliations:** 1 University Medical Unit, Teaching Hospital Batticaloa, Batticaloa, LKA; 2 Department of Clinical Sciences, Faculty of Health-Care Sciences, Eastern University of Sri Lanka, Batticaloa, LKA

**Keywords:** amlodipine, calcium channel blocker, hyperinsulinemiceuglycemic therapy, toxicity, type 2 respiratory failure

## Abstract

Amlodipine, a calcium channel blocker of the dihydropyridine class, is frequently used to treat high blood pressure. While overdoses are rare, they can result in significant cardiovascular compromise and, infrequently, respiratory failure. We report the case of a 17-year-old male patient who presented with an intentional amlodipine overdose, and his clinical course was complicated by type 2 (hypercapnic) respiratory failure, necessitating mechanical ventilation and intensive care management. He achieved full recovery with fluid resuscitation, calcium supplementation, high-dose insulin euglycemic therapy, vasopressor support, lung-protective ventilation, and supportive therapy. This case report highlights the importance of early detection and timely management of amlodipine toxicity, which can lead to severe complications such as cardiovascular instability, fluid overload, and respiratory distress with type 2 respiratory failure.

## Introduction

Amlodipine belongs to the dihydropyridine class of calcium channel blockers and is widely utilized in the treatment of high blood pressure and angina. Its effects are due to its selective action on L-type calcium channels in vascular smooth muscle. Overdose, though uncommon, can lead to profound hypotension, bradycardia, and, in severe cases, multiorgan dysfunction [1]. While the cardiovascular effects are well documented, complications such as respiratory failure are less frequently reported [2]. Type 2 respiratory failure (characterized by hypercapnia and alveolar hypoventilation) in the context of amlodipine toxicity may arise secondary to a combination of hemodynamic compromise, reduced respiratory drive, or secondary neuromuscular effects [3]. Early recognition and comprehensive supportive care are pivotal for a favorable outcome. Here, we present a case of amlodipine overdose in a young patient complicated by type 2 respiratory failure, successfully managed in an intensive care setting with early elective intubation.

## Case presentation

A 17-year-old boy was taken to the emergency department around four hours after intentionally ingesting approximately 60 tablets of amlodipine (5 mg each). He reported lightheadedness, nausea, and increasing shortness of breath. There was no significant past medical history, and no known allergies were reported. Initially, the patient was admitted to his local hospital, where he underwent gastric lavage, was given 50 g of activated charcoal and adequate hydration, and commenced on noradrenaline. He was later transferred to our hospital for further management.

**Figure 1:**
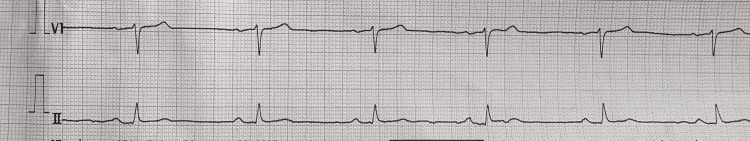
Electrocardiogram shows sinus bradycardia on the rhythm strip

**Figure 2:**
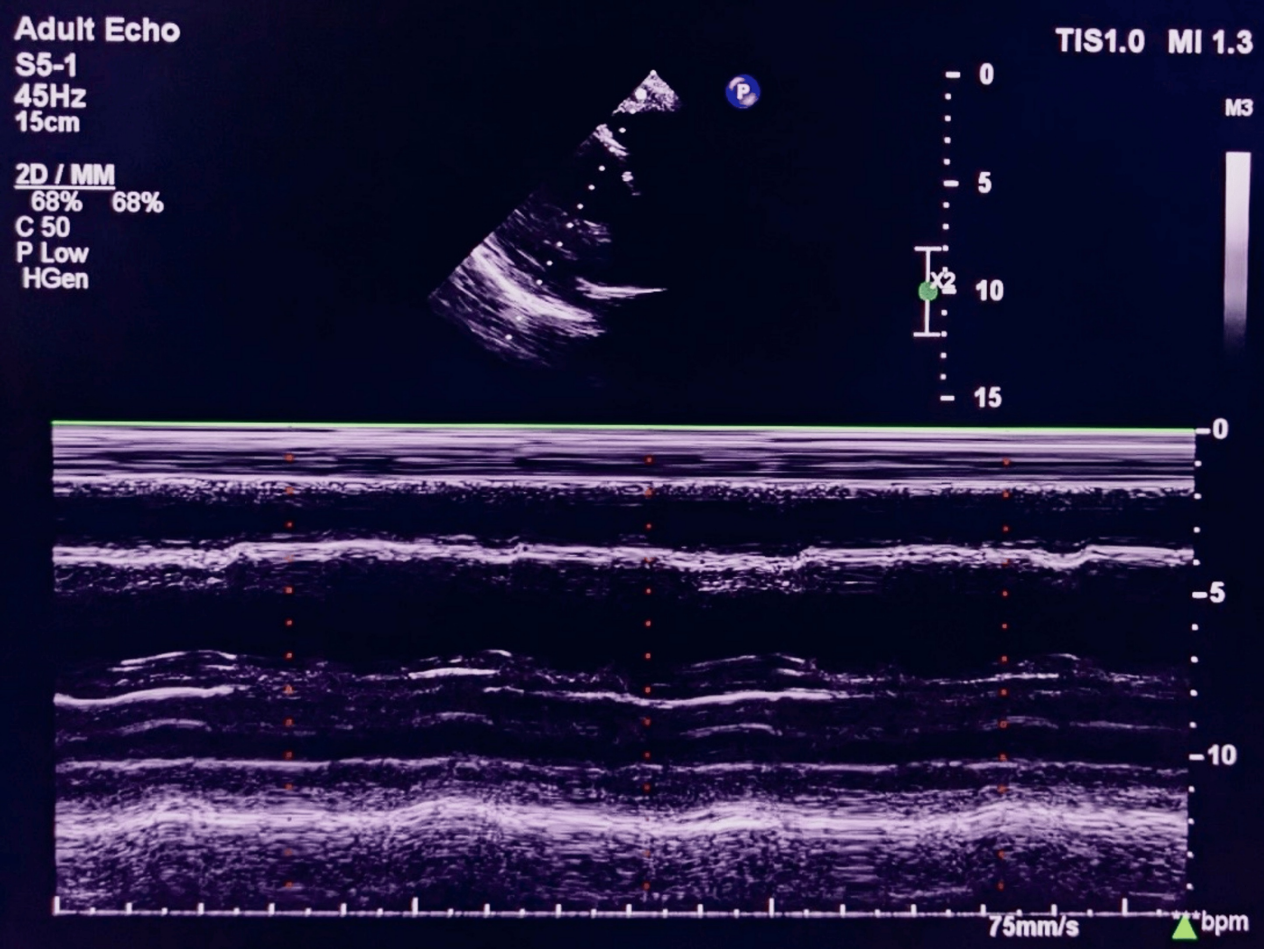
Transthoracic M-mode echocardiogram shows increased end-diastolic and end-systolic left ventricular diameters with global hypokinesia (HFrEF-35%) HFrEF: Heart failure with reduced ejection fraction

**Figure 3:**
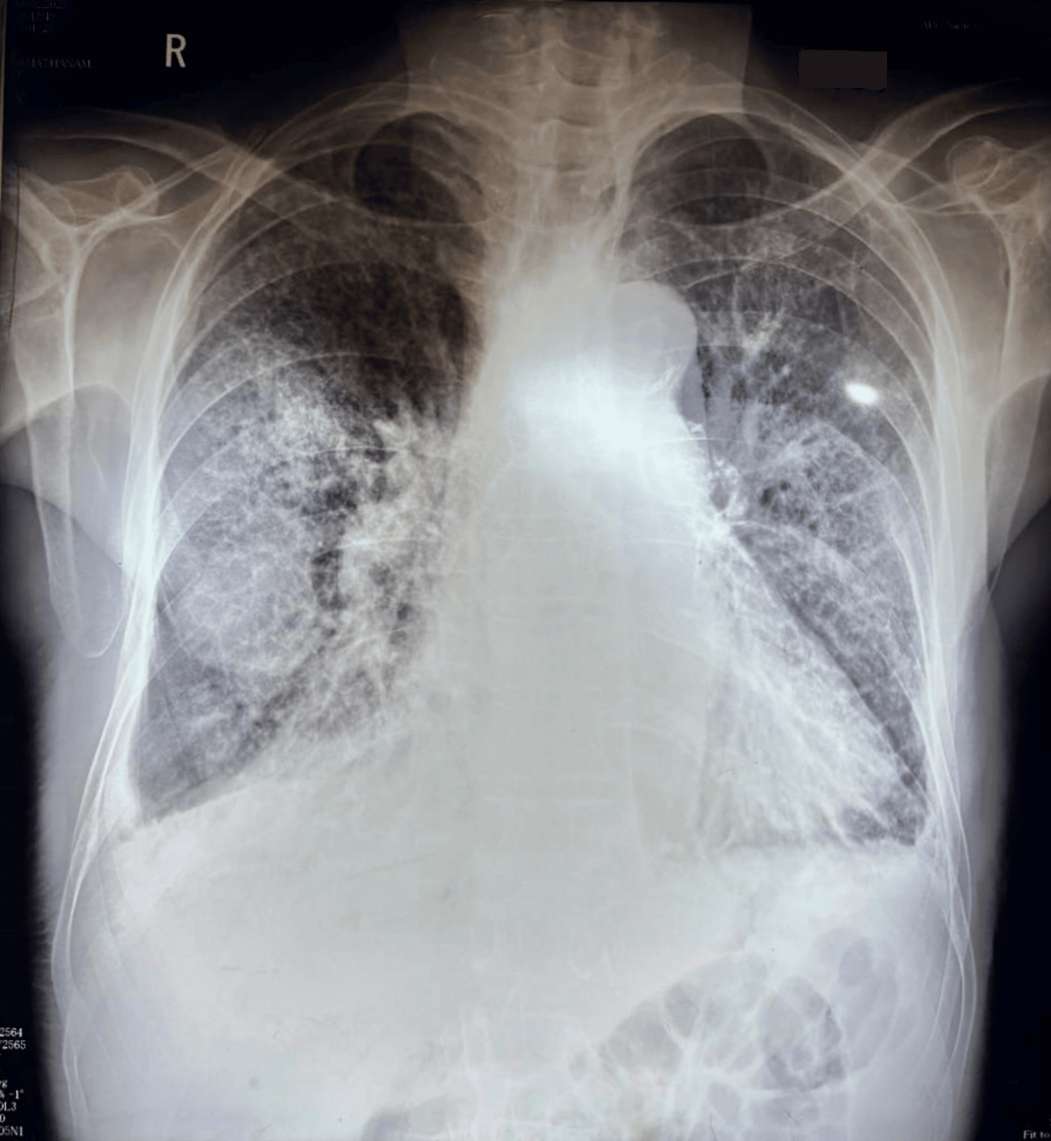
Chest X-ray shows alveolar edema with "Batwing" appearance. There were Kerley B lines and peribronchial cuffing due to interstitial edema. There were upper lobe diversion of pulmonary vessels, cardiomegaly, and bilateral blunting of costophrenic angles (pleural effusion) due to heart failure

On admission, the patient was conscious and afebrile at the resuscitation unit, with a pulse rate of 58 beats/minute, blood pressure of 90/50 mmHg, respiratory rate of 12 breaths/minute, and SpO2 of 96% on room air. On systemic examination, bilateral fine crepitations were heard on lung auscultation. Electrocardiography revealed sinus bradycardia without conduction blocks (Figure 1). Transthoracic echocardiography showed global hypokinesia of the left ventricle with an ejection fraction of 35% (Figure 2). Chest X-ray indicated features of pulmonary edema (Figure 3), while basic laboratory tests were otherwise normal.

High insulin euglycemic therapy (HIET) was started with an insulin-dextrose infusion (0.5 U/kg/hour) and a 10% calcium gluconate infusion (40 ml/hour). After an hour, his blood pressure dropped to 70/45 mmHg. Doses of noradrenaline were increased, and we added a dobutamine infusion. Despite these measures, the patient’s respiratory status deteriorated. The arterial blood gas analysis demonstrated worsening hypercapnia and hypoxemia consistent with type 2 respiratory failure. Because of the declining mental status and inadequate ventilation, the patient was intubated and placed on mechanical ventilation with lung-protective strategies. He was admitted to the intensive care unit, where he received continuous monitoring and supportive care. Insulin-dextrose and calcium gluconate infusions were gradually tapered off and stopped after 72 hours. On the fifth day, the patient was extubated, demonstrated complete clinical recovery, and was shifted to the ward. He was subsequently discharged on the seventh day, and outpatient follow-up, including counseling and further evaluation of his mental health, was arranged.

## Discussion

Amlodipine overdose predominantly manifests with cardiovascular depression, including hypotension and bradycardia, due to excessive inhibition of L-type calcium channels [4]. However, this case highlights that a profound overdose can also disrupt respiratory function, leading to type 2 respiratory failure characterized by inadequate alveolar ventilation and resultant hypercapnia [5,6]. This may occur secondary to central nervous system depression impairing respiratory drive, hemodynamic compromise reducing cardiac output, systemic hypotension leading to respiratory muscle fatigue, metabolic derangements from HIET, and the toxin itself transiently depressing neuromuscular function [7].

The management of calcium channel blocker toxicity remains largely supportive. The early institution of HIET has been associated with improved outcomes, likely by enhancing myocardial carbohydrate utilization and improving contractility [8]. Calcium supplementation helps overcome the competitive blockade at L-type channels, while vasopressors aid in maintaining perfusion [9]. In our patient, these therapies, coupled with timely respiratory support via mechanical ventilation, were critical in reversing the toxic effects and preventing further complications. In this case, we used a continuous intravenous infusion of 10% calcium gluconate at 40 ml/hour and insulin at 0.5 U/kg/hour with a 25% dextrose infusion at 50 ml/hour.

Atropine is administered for symptomatic bradycardia. If the bradycardia is severe and does not respond to atropine or an isoprenaline infusion, transvenous pacing is considered [10]. The primary concern is hypotension, which is first treated with intravenous fluids. After ensuring adequate hydration, inotropes may be introduced. Hemodialysis is not effective because these drugs are highly protein-bound, have a large volume of distribution (21 L/kg), and undergo rapid metabolism [11].

This case is a reminder that although respiratory failure is not the most common manifestation of amlodipine toxicity, clinicians should be vigilant for signs of hypoventilation and hypercapnia. A multidisciplinary approach involving critical care, toxicology, and mental health services is essential for the comprehensive management of such patients. While managing amlodipine toxicity can be difficult, timely and intensive treatment can significantly enhance the patient’s prognosis.

## Conclusions

This case report highlights the successful management of a 17-year-old male patient with an amlodipine overdose complicated by type 2 respiratory failure. Early aggressive supportive care, including IV fluids, calcium supplementation, high-dose insulin euglycemic therapy, vasopressor support, and timely mechanical ventilation, was pivotal in achieving a favorable outcome. Awareness of the potential for respiratory complications resulting from a calcium channel blocker overdose can facilitate prompt diagnosis and treatment, ultimately improving patient prognosis.
